# Gibbs measures of an Ising-Vannimenus Model with one-level competing interactions on 4th order Cayley tree

**DOI:** 10.1016/j.heliyon.2023.e13418

**Published:** 2023-02-07

**Authors:** Sanabel M. Abu Oun, Saed Mallak, Jihad Asad

**Affiliations:** a Department of Applied Mathematics, Faculty of Applied Sciences, Palestine Technical University- Kadoorie, Tulkarm P 305, West Bank, Palestine; b Department of Physics, Faculty of Applied Sciences, Palestine Technical University- Kadoorie, Tulkarm P 305, West Bank, Palestine

**Keywords:** Cayley tree, Gibbs measures, Ising- Vannimenus model, Phase transition

## Abstract

In this paper, we study an Ising-Vannimenus model on a fourth-order semi-infinite Cayley tree with one-level next-nearest-neighbor interactions. We construct the set of translation-invariant Gibbs measures corresponding to the model satisfying Kolmogorov consistency conditions. We prove the existence of the phase transition under specific conditions on the coupling constants and temperature. Numerical examples are presented to clarify the role of the added one-level interactions and results.

## 1 Introduction

Gibbs measure and infinite random systems were introduced in 1960's by Dobrushin who introduced the Markov random fields on the d-dimensional $Z^{d}$ lattices [1]. During the last few decades many authors dealt with the problem of Gibbs measures and phase transitions [[2], [3], [4], [5], [6], [7], [8], [9], [10], [11], [12], [13], [14], [15], [16], [17], [18], [19], [20], [21], [22], [23], [24], [25], [26], [27], [28], [29], [30], [31], [32], [33], [34], [35], [36], [37], [38], [39], [40]].

Bethe lattice (or Cayley tree) is not a realistic lattice, it was introduced in the physical literature in 1935 by the physicist Hans Bethe [18]. Many authors have considered the well-known models, the Ising and Potts models, on the Cayley tree [[1], [2], [3], [4], [5], [6], [7], [8], [9], [10], [11], [12], [13], [14], [15], [16], [17], [18], [19], [20], [23], [24], [25], 30, [34], [35], [36], [37], [38], [39], [40]].

In 2016, Akin described a specific set of Gibbs measures with a memory length equals to 2, corresponding to the Ising-Vannimenus model on a second-order Cayley tree using a new theoretical approach [5]. In 2017, he dealt with new Gibbs measures of the Vannimenus model with competing nearest-neighbor and prolonged next-nearest-neighbor interactions on a third-order Cayley tree. He described the set of translation-invariant Gibbs states for this model using Markov Random Field (MRF) approach [4]. In 2018, he generalized his results in Ref. [4] to an arbitrary order Cayley tree [3].

More complicated models should be studied on trees with the hope of discovering new phases or unusual types of behavior. In this paper, we consider the translation-invariant Gibbs measures with memory length equals to 2, corresponding to the Ising-Vannimenus model on the fourth-order Cayley tree with one-level next-nearest-neighbor interactions. We use the MRF method to construct the set of recurrence equations corresponding to the model. i.e., we turn the problem into nonlinear recursive relations along the branches of a Cayley tree of order four by fulfilling the Kolmogorov consistency conditions [2]. After that, we describe the translation-invariant Gibbs measures corresponding to the model and prove the existence of phase transitions. This is done by showing that there exist more than one extreme Gibbs distribution for specific values of the temperature and the coupling constants. Finally, we give a numerical example and explain the role of the added one-level interaction. Our model is a generalization of Akin's work [3] for Cayley tree of order four by adding one-level next-nearest-neighbor interaction.

## 2 Preliminaries

Definition 1 Consider a countably infinite set S and any measurable space (Φ,E). Then, the Random field (or a spin system) is a family ${\left(\varphi \left(x\right)\right)}_{x\in S}$ of random variables that are defined on some probability space and take its values in Φ. S is called the parameter set, Φ is the state space or spin space, and φ(x) is called the spin at state x. Let $\Omega =\Phi^{S}=\left\{{\left(\varphi \left(x\right)\right)}_{x\in S}:\varphi \left(x\right)\in \Phi \right\}\text{,}$ then Ω is called the set of all possible configurations, and the element σ∈ Ω is called a configuration [31].

**Fig. 1:**
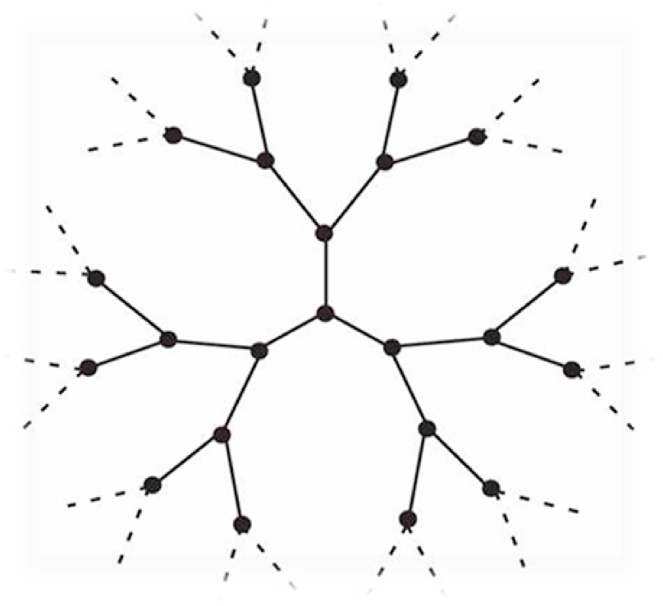
Second-order Cayley tree.

**Fig. 2:**
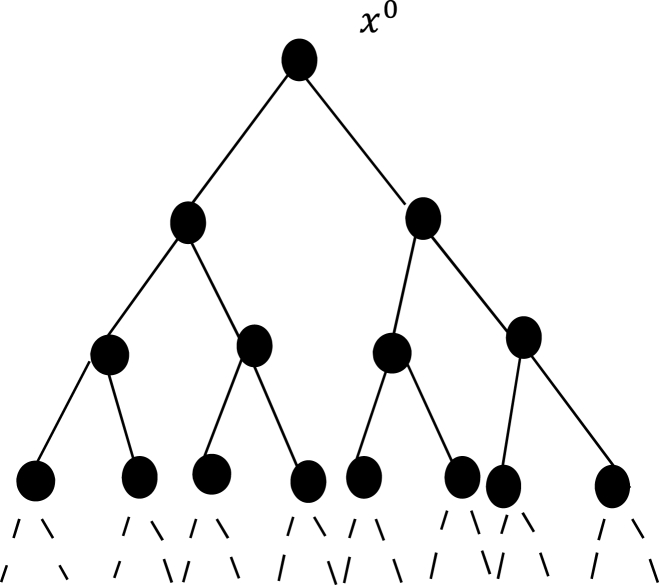
Second-order semi-infinite Cayley tree.

Definition 2 A regular Cayley tree of k ^th^-order $\left(\Gamma^{k},k\geq 1\right)$ is an infinite tree, i.e., a graph without cycles in which just k+1 edges emanate from each vertex. It is denoted as $\Gamma^{k}=\left(V,L\right)$, where V is the set of vertices and L is the set of edges of $\Gamma^{k}$. The semi-infinite k ^th^-order Cayley tree $\left(\Gamma_{+}^{k}\right)$ is an infinite graph without cycles in which only k edges emanate from the root vertex $x^{0}$ but k+1 edges issuing from any other vertex, see Fig. 1, Fig. 2 [22].

The number of edges in the smallest path between any two vertices $x,x^{\left(1\right)}\in V$ on the Cayley tree $\Gamma^{k}$ is called the distance from x to $x^{\left(1\right)}$ and is denoted by $d\left(x,x^{\left(1\right)}\right)\text{.}$

We denote the sphere of radius n on V by $W_{n}$ where:

$$W_{n}=\left\{x\in V:d\left(x,x^{0}\right)=n\right\}\text{.}$$

The vertices on $W_{n}$ are called the $n^{th}$ level.

If $x\in W_{n}$, then the vertices of the set $S\left(x\right)=\left\{x^{\left(i\right)}\in W_{n+1}:d\left(x,x^{\left(i\right)}\right)=1,i=1,\ldots ,k\right\}$ are called the direct successors of the vertex x. The ball of radius n on V is denoted by $V_{n}$ where: $V_{n}=\left\{x\in V:d\left(x,x^{0}\right)\leq n\right\}$ and $L_{n}$ is the set of edges in $V_{n}$.

For simplicity, we can put $\left|x\right|=d\left(x,x^{0}\right),x\in V\text{.}$

Two vertices $x,x^{\left(1\right)}\in V$ are called nearest-neighbors (NN) if they are connected by an edge l∈L, that is usually denoted by $l=<\;x,x^{\left(1\right)}\;>\text{.}$ These vertices are called next-nearest-neighbors (NNN) if $d\left(x,x^{\left(1\right)}\right)=2\text{.}$ The NNN vertices $x,x^{\left(1\right)}$ are termed prolonged (PNNN) if $\left|x\right|\neq \left|x^{\left(1\right)}\right|$ and denoted by $>x,x^{\left(1\right)}<$, but they are termed one-level next-nearest- neighbor (OLNNN) if $\left|x\right|=\left|x^{\left(1\right)}\right|$ and denoted by $<\hat{x,x^{\left(1\right)}}>$ [2].

Ising Model on Cayley tree is a model with spins that takes its values in the state space Φ={−1,1} located at the vertices of the Cayley tree. Define a configuration σ on V as a function such that σ:x∈V→σ(x)∈Φ and denote the set of all possible configurations by $\Phi^{V}$. Then the Hamiltonian for this model is defined by Eq [1].:

(1) $$H\left(\sigma \right)=-J\;\sum_{<x,x^{\left(1\right)}>\subset L}\sigma \left(x\right)\sigma \left(x^{\left(1\right)}\right)$$

where the sum is taken over the NN vertices $<\;x,x^{\left(1\right)}\;>$, [4] and [17].

In [11,12], the authors have considered Gibbs measures with memory two for Ising-Vannimenus models on a second-order Cayley tree. The vector-valued function is defined by:

$$h:<\;x,x^{\left(1\right)}\;>\to \;h_{xx^{\left(1\right)}}=\left(h_{xx^{\left(1\right)},++},h_{xx^{\left(1\right)},+-},h_{xx^{\left(1\right)},-+},h_{xx^{\left(1\right)},--}\right)\in R^{4}\text{,}$$

where $h_{xx^{\left(1\right)},\sigma \left(x\right)\sigma \left(x^{\left(1\right)}\right)}\in R$ and $x\in W_{n-1},x^{\left(1\right)}\in S(x$).

The (finite-dimensional) probability distribution of a Gibbs measure μ defined in the ball $V_{n}$ is the measure:

$$\mu_{n}=\frac{1}{Z_{n}}\;\exp\left[-\beta \;H_{n}\left(\sigma_{n}\right)+\sum_{x\in W_{n-1}}\sum_{x^{\left(1\right)}\in W_{n}}\sigma \left(x\right)\sigma \left(x^{\left(1\right)}\right)h_{xx^{\left(1\right)},\sigma \left(x\right)\sigma \left(x^{\left(1\right)}\right)}\right]$$

where $\beta =\frac{1}{kT}$ is called the inverse temperature and $Z_{n}$ is the partition function:

$$Z_{n}=\sum_{\sigma_{n}\in \Phi^{V_{n}}}\exp\left[-\beta \;H_{n}\left(\sigma_{n}\right)+\sum_{x\in W_{n-1}}\sum_{x^{\left(1\right)}\in W_{n}}\sigma \left(x\right)\sigma \left(x^{\left(1\right)}\right)h_{xx^{\left(1\right)},\sigma \left(x\right)\sigma \left(x^{\left(1\right)}\right)}\right]\text{.}$$

In [5], Akin defined the vector valued function by:

$h:<\;x,x^{\left(1\right)},x^{\left(2\right)}>\to \;h_{xx^{\left(1\right)}x^{\left(2\right)}}=\left(\tau_{1},\tau_{2},\tau_{3},\tau_{4},\tau_{5},\tau_{6},\tau_{7},\tau_{8}\right)\in R^{8}$, where:

$${\tau_{1}=h}_{xx^{\left(1\right)}x^{\left(2\right)},+++},\tau_{2}=h_{xx^{\left(1\right)}x^{\left(2\right)},+-+},\tau_{3}=h_{xx^{\left(1\right)}x^{\left(2\right)},++-},\tau_{4}=h_{xx^{\left(1\right)}x^{\left(2\right)},+--}$$

$${\tau_{5}=h}_{xx^{\left(1\right)}x^{\left(2\right)},-++},{\tau_{6}=h}_{xx^{\left(1\right)}x^{\left(2\right)},--+},\tau_{7}=h_{xx^{\left(1\right)}x^{\left(2\right)},-+-},{\tau_{8}=h}_{xx^{\left(1\right)}x^{\left(2\right)},---}$$

$$x\in W_{n-1},x^{\left(1\right)},x^{\left(2\right)}\in S\left(x\right)\;and\;h_{xx^{\left(1\right)}x^{\left(2\right)},\sigma \left(x\right)\sigma \left(x^{\left(1\right)}\right)\sigma \left(x^{\left(2\right)}\right)}\in R\text{.}$$

## 3 The model and recurrent equations

The general structure of Gibbs measure with memory length equals two on the 4th-order Cayley tree is presented as in [[2], [3], [4]]. Consider the Hamiltonian for Ising-Vannimenus model with OLNNN interaction. Therefore, the Hamiltonian is written in Eq [2]. below:

(2) $$H\left(\sigma \right)=-J_{oL}\sum_{\hat{<x,x^{\left(1\right)}>}}\sigma \left(x\right)\sigma \left(x^{\left(1\right)}\right)-J_{p}\sum_{>x,x^{\left(1\right)}<}\sigma \left(x\right)\sigma \left(x^{\left(1\right)}\right)-J\;\sum_{<x,x^{\left(1\right)}>}\sigma \left(x\right)\sigma \left(x^{\left(1\right)}\right)$$

Thus, our model defines the Ising-Vannimenus model with competing NN, PNNN and OLNNN interactions, where the sum in the first term ranges all One-Level NNN interactions. Here, the coupling constant $J_{oL}\in R$ is corresponding to OLNNN potentials. Briefly, this model is a development of Akin's work [3] on 4th-order Cayley tree by adding OLNNN interactions.

Let us consider a 4th-order Cayley tree. Let $x\in W_{n}$ for some n∈N and:

$$S\left(x\right)=\left\{x^{\left(1\right)},x^{\left(2\right)},x^{\left(3\right)},x^{\left(4\right)}\right\}$$

where $x^{\left(1\right)},x^{\left(2\right)},x^{\left(3\right)},x^{\left(4\right)}$ $\in W_{n+1}$ are the direct successors of x.

Denote $B_{1}\left(x\right)=\left\{x,x^{\left(1\right)},x^{\left(2\right)},x^{\left(3\right)},x^{\left(4\right)}\right\}$ a unit semi-ball with center x.

The set of all configurations on unit semi-ball $B_{1}\left(x\right)$ on Cayley tree of order four can be denoted by:

$\Phi^{B_{1}\left(x\right)}=\left\{\left(\begin{array}{c} j\\ l,m,p,q \end{array}\right):j,l,m,p,q\in \Phi \right\}.$It's clear that the set $\Phi^{B_{1}\left(x\right)}$ consists of 2^5^ [31] possible configurations [4].

Table 1 below shows the set of all possible configurations belonging to $\Phi^{B_{1}\left(x\right)}$. For simplicity, we assume that all branches of the Cayley tree are equivalent.

**Table 1:** The set of possible configurations ($\Phi^{B_{1}\left(x\right)})$ on 4th-order Cayley tree.

${\sigma_{1}}^{\left(1\right)}=\left(+,{+}_{,}^{+}+,+\right)$	${\sigma_{2}}^{\left(1\right)}$ = $\left(-,{+}_{,}^{+}+,+\right)$	${\sigma_{3}}^{\left(1\right)}$ = $\left(+,{-}_{,}^{+}+,+\right)$
${\sigma_{4}}^{\left(1\right)}=\left(+,{+}_{,}^{+}-,+\right)$	${\sigma_{5}}^{\left(1\right)}$ = $\left(+,{+}_{,}^{+}+,-\right)$	${\sigma_{6}}^{\left(1\right)}=\left(-,{-}_{,}^{+}+,+\right)$
${\sigma_{7}}^{\left(1\right)}=\left(-,{+}_{,}^{+}-,+\right)$	${\sigma_{8}}^{\left(1\right)}=\left(+,{-}_{,}^{+}+,-\right)$	${\sigma_{9}}^{\left(1\right)}=\left(-,{+}_{,}^{+}+,-\right)$
${\sigma_{10}}^{\left(1\right)}=\left(+,{-}_{,}^{+}-,+\right)$	${\sigma_{11}}^{\left(1\right)}=\left(+,{+}_{,}^{+}-,-\right)$	${\sigma_{12}}^{\left(1\right)}=\left(+,{-}_{,}^{+}-,-\right)$
${\sigma_{13}}^{\left(1\right)}=\left(-,{+}_{,}^{+}-,-\right)$	${\sigma_{14}}^{\left(1\right)}$ = $\left(-,{-}_{,}^{+}+,-\right)$	${\sigma_{15}}^{\left(1\right)}=\left(-,{-}_{,}^{+}-,+\right)$
${\sigma_{16}}^{\left(1\right)}$ = $\left(-,{-}_{,}^{+}-,-\right)$	${\sigma_{17}}^{\left(1\right)}$ = $\left(+,{+}_{,}^{-}+,+\right)$	${\sigma_{18}}^{\left(1\right)}=\left(-,{+}_{,}^{-}+,+\right)$
${\sigma_{19}}^{\left(1\right)}$ = $\left(+,{-}_{,}^{-}+,+\right)$	${\sigma_{20}}^{\left(1\right)}$ = $\left(+,{+}_{,}^{-}-,+\right)$	${\sigma_{21}}^{\left(1\right)}=\left(+,{+}_{,}^{-}+,-\right)$
${\sigma_{22}}^{\left(1\right)}$ = $\left(-,{-}_{,}^{-}+,+\right)$	${\sigma_{23}}^{\left(1\right)}$ = $\left(-,{+}_{,}^{-}-,+\right)$	${\sigma_{24}}^{\left(1\right)}$ = $\left(+,{-}_{,}^{-}+,-\right)$
${\sigma_{25}}^{\left(1\right)}=\left(-,{+}_{,}^{-}+,-\right)$	${\sigma_{26}}^{\left(1\right)}$ = $\left(+,{-}_{,}^{-}-,+\right)$	${\sigma_{27}}^{\left(1\right)}$ = $\left(+,{+}_{,}^{-}-,-\right)$
${\sigma_{28}}^{\left(1\right)}=\left(+,{-}_{,}^{-}-,-\right)$	${\sigma_{29}}^{\left(1\right)}$ = $\left(-,{+}_{,}^{-}-,-\right)$	${\sigma_{30}}^{\left(1\right)}=\left(-,{-}_{,}^{-}+,-\right)$
${\sigma_{31}}^{\left(1\right)}=\left(-,{-}_{,}^{-}-,+\right)$	${\sigma_{32}}^{\left(1\right)}=\left(-,{-}_{,}^{-}-,-\right)$	

In Eq [3]. we consider the following definition for the vector valued function $h:V\;\to R^{32}$:

(3) $$\begin{array}{c} h:<x,x^{\left(1\right)},x^{\left(2\right)},x^{\left(3\right)},x^{\left(4\right)}>\;\to h_{B_{1}\left(x\right)}=\left(h_{B_{1}\left(x\right),\sigma \left(x\right)\sigma \left(x^{\left(1\right)}\right)\sigma \left(x^{\left(2\right)}\right)\sigma \left(x^{\left(3\right)}\right)\sigma \left(x^{\left(4\right)}\right)}:\sigma \left(x\right),\sigma \left(x^{\left(1\right)}\right),\sigma \left(x^{\left(2\right)}\right),\sigma \left(x^{\left(3\right)}\right),\sigma \left(x^{\left(4\right)}\right)\in \Phi \right) \end{array}$$

where, $h_{B_{1}\left(x\right),\sigma \left(x\right)\sigma \left(x^{\left(1\right)}\right)\sigma \left(x^{\left(2\right)}\right)\sigma \left(x^{\left(3\right)}\right)\sigma \left(x^{\left(4\right)}\right)}\in R$, $x\in W_{n-1}$, $x^{\left(1\right)},x^{\left(2\right)},x^{\left(3\right)},x^{\left(4\right)}\in S\left(x\right)$ and $<x,x^{\left(1\right)},x^{\left(2\right)},x^{\left(3\right)},x^{\left(4\right)}>$ denotes the vertices for the semi ball $B_{1}\left(x\right)$.

We use the function $h_{B_{1}\left(x\right),\sigma \left(x\right)\sigma \left(x^{\left(1\right)}\right)\sigma \left(x^{\left(2\right)}\right)\sigma \left(x^{\left(3\right)}\right)\sigma \left(x^{\left(4\right)}\right)}$ to describe the Gibbs measure of any configuration $\left(\begin{array}{l} \sigma \left(x\right)\\ \sigma \left(x^{\left(1\right)}\right)\;\sigma \left(x^{\left(2\right)}\right)\;\sigma \left(x^{\left(3\right)}\right)\;\sigma \left(x^{\left(4\right)}\right) \end{array}\right)$ that belongs to $\Phi^{B_{1}\left(x\right)}$.

### 3.1 Gibbs measures construction

The finite-dimensional Gibbs probability measures on the configuration space $\Phi^{V_{n}}$ (the set of all possible configurations on the ball $V_{n}$) at inverse temperature $\beta =\frac{1}{kT}$ on 4th-order Cayley tree is defined by the formula given in Eq [4].:

(4) $${\mu_{h}}^{\left(n\right)}\left(\sigma \right)=\frac{1}{Z_{n}}\exp\left[-\beta \;H_{n}\left(\sigma \right)+\varrho \right]$$

where $\varrho =\sum_{x\in w_{n-1}}\sum_{x^{\left(1\right)},x^{\left(2\right)},x^{\left(3\right)},x^{\left(4\right)}\in S\left(x\right)}\sigma \left(x\right)\sigma \left(x^{\left(1\right)}\right)\sigma \left(x^{\left(2\right)}\right)\sigma \left(x^{\left(3\right)}\right)\sigma \left(x^{\left(4\right)}\right)\varrho_{1}$

$${\varrho_{1}=h}_{B_{1}\left(x\right),\sigma \left(x\right)\sigma \left(x^{\left(1\right)}\right)\sigma \left(x^{\left(2\right)}\right)\sigma \left(x^{\left(3\right)}\right)\sigma \left(x^{\left(4\right)}\right)}$$

and $Z_{n}$ is the corresponding partition function defined as:

$$Z_{n}=\sum_{\sigma_{n}\in \Phi^{V_{n}}}\exp\left[-\beta \;H_{n}\left(\sigma \right)+\varrho \right]\text{.}$$

Now we are interested in the construction of an infinite volume distribution (limiting Gibbs measure) from the given finite-dimensional Gibbs distributions. The limiting Gibbs measures that we look for are the set of translation-invariant ones.

As in Refs. [[3], [4], [5]], a finite dimensional measures ${\mu_{h}}^{\left(n\right)}$ are compatible (consistent) if for all n≥1 and $\sigma_{n-1}\in \Phi^{V_{n-1}}\text{:}$

$$\sum_{w\in \Phi^{w_{n}}}\mu_{n}(\sigma_{n-1}\vee w)=\mu_{n-1}(\sigma_{n-1})$$

where $\sigma_{n-1}\vee w$ is the concatenation of the configurations, i.e.:

if $x\in V_{n-1}\text{,}$ then $\left(\sigma_{n-1}\vee w\right)\;\left(x\right)=\sigma_{n-1}\left(x\right)$, while if $x\in W_{n}$, then $\left(\sigma_{n-1}\vee w\right)\;\left(x\right)=w\left(x\right)$.

If this condition is satisfied, then there exists a unique limiting Gibbs measure $\mu_{h}$ such that:

$$\mu_{h}\left(\sigma \in \Omega :\sigma {|}_{V_{n}}=\sigma_{n}\right)=\mu_{h}^{\left(n\right)}\left(\sigma_{n}\right),for\;all\;\sigma_{n}\in \Phi^{V_{n}},n\in N\text{.}$$

Firstly, we try to find the set of limiting Gibbs measures $\mu_{h}$ with a memory length 2 for the sequence of finite-dimensional compatible measures that correspond to the Hamiltonian of our model [2] and the boundary function h [3].

We define the Hamiltonian (i.e., Eq. [5]) on V with the configuration $\sigma_{n-1}\in \Phi^{V_{n-1}}$ on the inner ball $V_{n-1}$ and the boundary condition $w\in \Phi^{W_{n}}$ as:

(5) $$\begin{array}{c} H_{n}\left(\sigma_{n-1}\vee w\right)=\\ -J_{oL}\sum_{\begin{array}{l} \;\\ \hat{<x,x^{\left(1\right)}>}{\in V}_{n-1} \end{array}}\sigma \left(x\right)\sigma \left(x^{\left(1\right)}\right)-J_{oL}\sum_{\begin{array}{l} \hat{<x,x^{\left(1\right)}>}\;{\in W}_{n} \end{array}}w\left(x\right)w\left(x^{\left(1\right)}\right)\\ -J_{p}\sum_{>x,x^{\left(1\right)}<\;{\in V}_{n-1}}\sigma \left(x\right)\sigma \left(x^{\left(1\right)}\right)-J_{p}\sum_{x\in W_{n-2}}\sum_{x^{\left(1\right)}\in S^{2}\left(x\right)}\sigma \left(x\right)w\left(x^{\left(1\right)}\right)\\ -J\sum_{<x,x^{\left(1\right)}>\;{\in V}_{n-1}}\sigma \left(x\right)\sigma \left(x^{\left(1\right)}\right)-J\sum_{x\in W_{n-1}}\sum_{x^{\left(1\right)}\in S\left(x\right)}\sigma \left(x\right)w\left(x^{\left(1\right)}\right)\\ =H\left(\sigma_{n-1}\right)-J_{oL}\sum_{\hat{<x,x^{\left(1\right)}>}\in W_{n}}w\left(x\right)w\left(x^{\left(1\right)}\right)-J_{p}\sum_{x\in W_{n-2}}\sum_{x^{\left(1\right)}\in S^{2}\left(x\right)}\sigma \left(x\right)w\left(x^{\left(1\right)}\right)\\ -J\sum_{x\in W_{n-1}}\sum_{x^{\left(1\right)}\in S\left(x\right)}\sigma \left(x\right)w\left(x^{\left(1\right)}\right). \end{array}$$

Thus, the compatibility condition is satisfied for the sequence of finite dimensional probability measures ${\mu_{h}}^{\left(n\right)},n\geq 1$ that we have mentioned in Ref. [4] if the equality in Eq [6]. is satisfied

(6) $$\sum_{w\in \Phi^{w_{n}}}\mu_{h}^{\left(n\right)}(\sigma_{n-1}\vee w)=\mu_{h}^{\left(n-1\right)}(\sigma_{n-1})$$

then we get:

$$\frac{1}{z_{n}}\;\sum_{w\in \Phi^{W_{n}}}\;\exp\left[-\beta H_{n}\left(\sigma_{n-1}\vee w\right)+\sum_{x^{\left(1\right)},x^{\left(2\right)},x^{\left(3\right)},x^{\left(4\right)}\in W_{n-1}}\sum_{x_{i}^{\left(1\right)}\in S\left(x^{\left(1\right)}\right)}\sum_{x_{i}^{\left(2\right)}\in S\left(x^{\left(2\right)}\right)}\sum_{x_{i}^{\left(3\right)}\in S\left(x^{\left(3\right)}\right)}\sum_{x_{i}^{\left(4\right)}\in S\left(x^{\left(4\right)}\right)}\Theta \right]$$

$$=\frac{1}{z_{n-1}}\exp\left[-\beta H_{n}\left(\sigma_{n-1}\right)+\sum_{x\in W_{n-2}}\sum_{x^{\left(1\right)},x^{\left(2\right)},x^{\left(3\right)},x^{\left(4\right)}\in S\left(x\right)}\psi \right]$$

where $\Theta =\Theta_{1}+\Theta_{2}+\Theta_{3}+\Theta_{4}$

$$\begin{array}{c} \Theta_{1}=\sigma \left(x^{\left(1\right)}\right)\;w\left(x_{1}^{\left(1\right)}\right)\;w\left(x_{2}^{\left(1\right)}\right)\;w\left(x_{2}^{\left(1\right)}\right)\\ w\left(x_{2}^{\left(1\right)}\right)h_{B_{1}\left(x^{\left(1\right)}\right),\sigma \left(x^{\left(1\right)}\right)\;w\left(x_{1}^{\left(1\right)}\right)\;w\left(x_{2}^{\left(1\right)}\right)w\left(x_{3}^{\left(1\right)}\right)\;w\left(x_{4}^{\left(1\right)}\right)} \end{array}$$

$$\begin{array}{c} \Theta_{2}=\sigma \left(x^{\left(2\right)}\right)\;w\left(x_{1}^{\left(2\right)}\right)\;w\left(x_{2}^{\left(2\right)}\right)\;w\left(x_{3}^{\left(2\right)}\right)\\ {w\left(x_{4}^{\left(2\right)}\right)\;h}_{B_{1}\left(x^{\left(2\right)}\right),\sigma \left(x^{\left(2\right)}\right)\;w\left(x_{1}^{\left(2\right)}\right)\;w\left(x_{2}^{\left(2\right)}\right)w\left(x_{3}^{\left(2\right)}\right)\;w\left(x_{4}^{\left(2\right)}\right)} \end{array}$$

$$\begin{array}{c} \Theta_{3}=\sigma \left(x^{\left(3\right)}\right)\;w\left(x_{1}^{\left(3\right)}\right)\;w\left(x_{2}^{\left(3\right)}\right)\;w\left(x_{3}^{\left(3\right)}\right)\\ {w\left(x_{4}^{\left(3\right)}\right)\;h}_{B_{1}\left(x^{\left(3\right)}\right),\sigma \left(x^{\left(3\right)}\right)\;w\left(x_{1}^{\left(3\right)}\right)\;w\left(x_{2}^{\left(3\right)}\right)w\left(x_{3}^{\left(3\right)}\right)\;w\left(x_{4}^{\left(3\right)}\right)} \end{array}$$

$$\begin{array}{c} \Theta_{4}=\sigma \left(x^{\left(4\right)}\right)\;w\left(x_{1}^{\left(4\right)}\right)\;w\left(x_{2}^{\left(4\right)}\right)\;w\left(x_{3}^{\left(4\right)}\right)\\ {w\left(x_{4}^{\left(4\right)}\right)\;h}_{B_{1}\left(x^{\left(4\right)}\right),\sigma \left(x^{\left(4\right)}\right)\;w\left(x_{1}^{\left(4\right)}\right)\;w\left(x_{2}^{\left(4\right)}\right)w\left(x_{3}^{\left(4\right)}\right)\;w\left(x_{4}^{\left(4\right)}\right)} \end{array}$$

and ${\psi =\sigma \left(x\right)\;\sigma \left(x^{\left(1\right)}\right)\;\sigma \left(x^{\left(2\right)}\right)\;\sigma \left(x^{\left(3\right)}\right)\;\sigma \left(x^{\left(4\right)}\right)h}_{B_{1}\left(x\right),\sigma \left(x\right)\;\sigma \left(x^{\left(1\right)}\right)\;\sigma \left(x^{\left(2\right)}\right)\;\sigma \left(x^{\left(3\right)}\right)\;\sigma \left(x^{\left(4\right)}\right)}$

Let $L_{n}=\frac{Z_{n-1}}{Z_{n}}$. Simplifying [5] and for each i=1,2,3,4 we obtained Eq [7]. below:

(7) $$\begin{array}{c} \prod_{x\in W_{n-2}}\prod_{x^{\left(1\right)},x^{\left(2\right)},x^{\left(3\right)},x^{\left(4\right)}\in S\left(x\right)}e^{{\sigma \left(x\right)\;\sigma \left(x^{\left(1\right)}\right)\;\sigma \left(x^{\left(2\right)}\right)\;\sigma \left(x^{\left(3\right)}\right)\;\sigma \left(x^{\left(4\right)}\right)h}_{B_{1}\left(x\right),\sigma \left(x\right)\;\sigma \left(x^{\left(1\right)}\right)\;\sigma \left(x^{\left(2\right)}\right)\;\sigma \left(x^{\left(3\right)}\right)\;\sigma \left(x^{\left(4\right)}\right)}}\\ {=L}_{n}\;\prod_{x\in W_{n-2}}\prod_{x^{\left(1\right)},x^{\left(2\right)},x^{\left(3\right)},x^{\left(4\right)}\in S\left(x\right)}\prod_{x_{i}^{\left(1\right)}\in S\left(x^{\left(1\right)}\right)}\prod_{x_{i}^{\left(2\right)}\in S\left(x^{\left(2\right)}\right)}\prod_{x_{i}^{\left(3\right)}\in S\left(x^{\left(3\right)}\right)}\prod_{x_{i}^{\left(4\right)}\in S\left(x^{\left(4\right)}\right)}I \end{array}$$

where

$$I=\sum_{w\left(x_{i}^{\left(1\right)}\right),w\left(x_{i}^{\left(2\right)}\right),w\left(x_{i}^{\left(3\right)}\right),w\left(x_{i}^{\left(4\right)}\right)\in \left\{-1.+1\right\}}e^{\left[Q\left(h,J,J_{p},J_{oL}\right)\right]}$$

$$Q\left(h,J,J_{p},J_{oL}\right)={\sigma \left(x^{\left(1\right)}\right)\;w\left(x_{1}^{\left(1\right)}\right)\;w\left(x_{2}^{\left(1\right)}\right)\;w\left(x_{2}^{\left(1\right)}\right)\;w\left(x_{2}^{\left(1\right)}\right)h}_{B_{1}\left(x^{(1})\right),\sigma \left(x^{\left(1\right)}\right)\;w\left(x_{1}^{\left(1\right)}\right)\;w\left(x_{2}^{\left(1\right)}\right)w\left(x_{3}^{\left(1\right)}\right)\;w\left(x_{4}^{\left(1\right)}\right)}+{{\sigma \left(x^{\left(2\right)}\right)\;w\left(x_{1}^{\left(2\right)}\right)\;w\left(x_{2}^{\left(2\right)}\right)\;w\left(x_{3}^{\left(2\right)}\right)\;w\left(x_{4}^{\left(2\right)}\right)\;h}_{B_{1}\left(x^{\left(2\right)}\right),\sigma \left(x^{\left(2\right)}\right)\;w\left(x_{1}^{\left(2\right)}\right)\;w\left(x_{2}^{\left(2\right)}\right)w\left(x_{3}^{\left(2\right)}\right)\;w\left(x_{4}^{\left(2\right)}\right)}}_{++{\sigma \left(x^{\left(3\right)}\right)\;w\left(x_{1}^{\left(3\right)}\right)\;w\left(x_{2}^{\left(3\right)}\right)\;w\left(x_{3}^{\left(3\right)}\right)\;w\left(x_{4}^{\left(3\right)}\right)\;h}_{B_{1}\left(x^{\left(3\right)}\right),\sigma \left(x^{\left(3\right)}\right)\;w\left(x_{1}^{\left(3\right)}\right)\;w\left(x_{2}^{\left(3\right)}\right)w\left(x_{3}^{\left(3\right)}\right)\;w\left(x_{4}^{\left(3\right)}\right)}+{\sigma \left(x^{\left(4\right)}\right)\;w\left(x_{1}^{\left(4\right)}\right)\;w\left(x_{2}^{\left(4\right)}\right)\;w\left(x_{3}^{\left(4\right)}\right)\;w\left(x_{4}^{\left(4\right)}\right)\;h}_{B_{1}\left(x^{\left(4\right)}\right),\sigma \left(x^{\left(4\right)}\right)\;w\left(x_{1}^{\left(4\right)}\right)\;w\left(x_{2}^{\left(4\right)}\right)w\left(x_{3}^{\left(4\right)}\right)\;w\left(x_{4}^{\left(4\right)}\right)}+\beta \;J\;\left[\sigma \left(x^{\left(1\right)}\right)\left(w\left(x_{1}^{\left(1\right)}\right)+w\left(x_{2}^{\left(1\right)}\right)+w\left(x_{2}^{\left(1\right)}\right)+w\left(x_{2}^{\left(1\right)}\right)\right)+\sigma \left(x^{\left(2\right)}\right)\left(w\left(x_{1}^{\left(2\right)}\right)+w\left(x_{2}^{\left(2\right)}\right)+w\left(x_{3}^{\left(2\right)}\right)+w\left(x_{4}^{\left(2\right)}\right)\right)+\sigma \left(x^{\left(3\right)}\right)\left(w\left(x_{1}^{\left(3\right)}\right)+w\left(x_{2}^{\left(3\right)}\right)+w\left(x_{3}^{\left(3\right)}\right)+w\left(x_{4}^{\left(3\right)}\right)\right)+\sigma \left(x^{\left(4\right)}\right)\left(w\left(x_{1}^{\left(4\right)}\right)+w\left(x_{2}^{\left(4\right)}\right)+w\left(x_{3}^{\left(4\right)}\right)+w\left(x_{4}^{\left(4\right)}\right)\right)\right]+\beta \;J_{OL}\;\left(\prod_{i=1}^{4}w\left(x_{i}^{\left(1\right)}\right)+\prod_{i=1}^{4}w\left(x_{i}^{\left(2\right)}\right)+\prod_{i=1}^{4}w\left(x_{i}^{\left(3\right)}\right)+\prod_{i=1}^{4}w\left(x_{i}^{\left(4\right)}\right)\right)+\beta \;J_{p}\left[\sigma \left(x\right)\sum_{i=1}^{4}\left(w\left(x_{i}^{\left(1\right)}\right)+w\left(x_{i}^{\left(2\right)}\right)+w\left(x_{i}^{\left(3\right)}\right)+w\left(x_{i}^{\left(4\right)}\right)\right)\right]}$$

Next, we fix the configurations on the edges $<\;x,x^{\left(1\right)}\;>,<\;x,x^{\left(2\right)}\;>$, $<\;x,x^{\left(3\right)}\;>$ and $<\;x,x^{\left(4\right)}\;>$ , and then rewrite [6] for all possible values of $\sigma \left(x\right),\sigma \left(x^{\left(1\right)}\right),\sigma \left(x^{\left(2\right)}\right),\sigma \left(x^{\left(3\right)}\right),\sigma \left(x^{\left(4\right)}\right)\in \Phi$ in order to construct the recurrence equations.

**Fig. 3:**
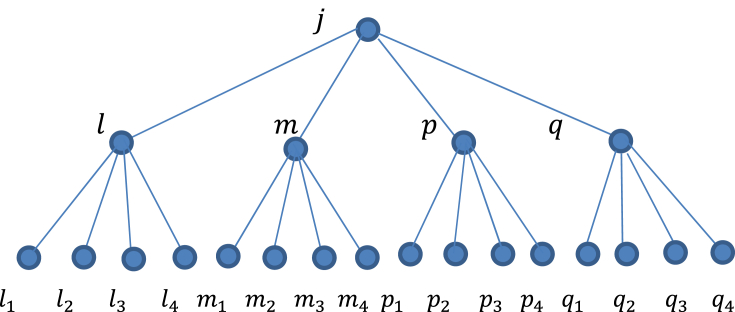
The set of configurations on 4th-order semi-infinite Cayley tree with 2 levels.

Assume $\sigma \left(x\right)=j,\sigma \left(x^{\left(1\right)}\right)=l,\sigma \left(x^{\left(2\right)}\right)=m,\sigma \left(x^{\left(3\right)}\right)=p,\sigma \left(x^{\left(4\right)}\right)=q,w\left(x_{i}^{\left(1\right)}\right)=l_{i},w\left(x_{i}^{\left(2\right)}\right)=m_{i},w\left(x_{i}^{\left(2\right)}\right)=m_{i},w\left(x_{i}^{\left(3\right)}\right)=p_{i}$ and $w\left(x_{i}^{\left(4\right)}\right)=q_{i}\text{.}$Where i=1,2,3,4 and $j,l,m,p,q,l_{i},m_{i},p_{i},q_{i}\in \Phi$. Then [6], is reduced to:

(8) $$e^{jlmp{q\;h}_{B_{1}\left(x\right),j;l,m,p,q}}=L_{2}\sum_{l_{1},m_{1},p_{1},\ldots .}\delta_{1}\times \delta_{2}\times \delta_{3}\times \delta_{4}\times \delta_{5}\times \delta_{6}$$

where

$$\delta_{1}=e^{\beta J_{p}j\left(l_{1}+l_{2}+l_{3}+l_{4}+m_{1}+m_{2}+m_{3}+m_{4}+P_{1}+P_{2}+P_{3}+P_{4}+q_{1}+q_{2}+q_{3}+q_{4}\right)}$$

$${\delta_{2}=e}^{\beta J\left(l\;\left(l_{1}+l_{2}+l_{3}+l_{4}\right)+m\;\left(m_{1}+m_{2}+m_{3}+m_{4}\right)+p\;\left(p_{1}+p_{2}+p_{3}+p_{4}\right)+q\;(q_{1}+q_{2}+q_{3}+q_{4}\right))}$$

$${\delta_{3}=e}^{\beta J_{0L}\left(\left(l_{1}\;\left(l_{2}+l_{3}+l_{4}\right)+l_{2}\left(l_{3}+l_{4}\right)+l_{3}l_{4}\right)\right)+\left(m_{1}\left(m_{2}+m_{3}+m_{4}\right)+m_{2}\left(m_{3}+m_{4}\right)+m_{3}m_{4}\right)}$$

$$\delta_{4}=e^{\beta J_{0L}(\;(p_{1}\left(p_{2}+p_{3}+p_{4})+p_{2}\left(p_{3}+p_{4}\right)+p_{3}\;p_{4}\right)+\left(q_{1}\left(q_{2}+q_{3}+q_{4}\right)+q_{2}\left(q_{3}+q_{4}\right)+q_{3}q_{4}\right))}$$

$$\delta_{5}{=e}^{l\;l_{1}l_{2}l_{3}l_{4}\;h_{B_{1}\left(x^{\left(1\right)}\right),l;l_{1},l_{2},l_{3},l_{4}}}\times e^{m\;{m_{1}m}_{2}{m_{3}m}_{4}\;h_{B_{1}\left(x^{\left(2\right)}\right),m;m_{1},m_{2},m_{3},m_{4}}}$$

$${\delta_{6}=e}^{p\;p_{1}p_{2}p_{3}p_{4}\;h_{B_{1}\left(x^{\left(3\right)}\right),p;p_{1},p_{2},p_{3},p_{4}}}\times e^{q\;{q_{1}q_{2}q_{3}q_{4}h}_{B_{1}\left(x^{\left(4\right)}\right),q;q_{1},q_{2},q_{3},q_{4}}}$$

and $L_{2}=\frac{Z_{1}}{Z_{2}}$ (Fig. 3).

As in [3,4], we define the real vector-valued function *h* given in (3) as in equations (9), (10), (11), (12), (13), (14), (15), (16), (17), (18), (19):

(9) $${h_{1}=h}_{B_{1}\left(x\right),{\sigma_{1}}^{\left(1\right)}}$$

(10) $${h_{2}=h}_{B_{1}\left(x\right),{\sigma_{2}}^{\left(1\right)}}=h_{B_{1}\left(x\right),{\sigma_{3}}^{\left(1\right)}}=h_{B_{1}\left(x\right),{\sigma_{4}}^{\left(1\right)}}=h_{B_{1}\left(x\right),{\sigma_{5}}^{\left(1\right)}}$$

(11) $$\begin{array}{c} {h_{3}=h}_{B_{1}\left(x\right),{\sigma_{6}}^{\left(1\right)}}=h_{B_{1}\left(x\right),{\sigma_{7}}^{\left(1\right)}}=h_{B_{1}\left(x\right),{\sigma_{8}}^{\left(1\right)}}=\\ h_{B_{1}\left(x\right),{\sigma_{9}}^{\left(1\right)}}=h_{B_{1}\left(x\right),{\sigma_{10}}^{\left(1\right)}=}h_{B_{1}\left(x\right),{\sigma_{11}}^{\left(1\right)}} \end{array}$$

(12) $${h_{4}=h}_{B_{1}\left(x\right),{\sigma_{12}}^{\left(1\right)}}=h_{B_{1}\left(x\right),{\sigma_{13}}^{\left(1\right)}}=h_{B_{1}\left(x\right),{\sigma_{14}}^{\left(1\right)}}=h_{B_{1}\left(x\right),{\sigma_{15}}^{\left(1\right)}}=h_{B_{1}\left(x\right),{\sigma_{15}}^{\left(1\right)}}$$

(13) $$h_{5}=h_{B_{1}\left(x\right),{\sigma_{16}}^{\left(1\right)}}$$

(14) $${h_{6}=h}_{B_{1}\left(x\right),{\sigma_{17}}^{\left(1\right)}}$$

(15) $$h_{7}=h_{B_{1}\left(x\right),{\sigma_{18}}^{\left(1\right)}}=h_{B_{1}\left(x\right),{\sigma_{19}}^{\left(1\right)}}=h_{B_{1}\left(x\right),{\sigma_{20}}^{\left(1\right)}}{=h}_{B_{1}\left(x\right),{\sigma_{21}}^{\left(1\right)}}$$

(16) $$h_{8}=h_{B_{1}\left(x\right),{\sigma_{22}}^{\left(1\right)}}=h_{B_{1}\left(x\right),{\sigma_{23}}^{\left(1\right)}}=h_{B_{1}\left(x\right),{\sigma_{24}}^{\left(1\right)}}=h_{B_{1}\left(x\right),{\sigma_{25}}^{\left(1\right)}}=h_{B_{1}\left(x\right),{\sigma_{26}}^{\left(1\right)}}{=h}_{B_{1}\left(x\right),{\sigma_{27}}^{\left(1\right)}}$$

(17) $$h_{9}=h_{B_{1}\left(x\right),{\sigma_{28}}^{\left(1\right)}}=h_{B_{1}\left(x\right),{\sigma_{29}}^{\left(1\right)}}=h_{B_{1}\left(x\right),{\sigma_{30}}^{\left(1\right)}}=h_{B_{1}\left(x\right),{\sigma_{31}}^{\left(1\right)}}$$

(18) $$h_{10}=h_{B_{1}\left(x\right),{\sigma_{32}}^{\left(1\right)}}.$$

Therefore, the real vector**-**valued function h can be redefined as:

(19) $$h\left(x\right)=\left(h_{1},h_{2},h_{3},h_{4},h_{5},h_{6},h_{7},h_{8},h_{9},h_{10}\right)\in R^{10},x\in W_{n-1},n\geq 1$$

### 3.2 Recurrence equations construction

Now, we construct the recurrence equations that give a formula to describe Gibbs measures with memory of length two which satisfies consistency conditions by means of equation (8).

Assume that $a=e^{\beta J}$, $b=e^{\beta J_{p}}$ and $c=e^{\beta J_{OL}}$. Let:

${v_{i}}^{\prime }=e^{h_{B_{1}\left(x\right),\sigma_{j}^{\left(1\right)}}}$ for $x\in W_{n-1}$ & $v_{i}=e^{h_{B_{1}\left(y\right),\sigma_{j}^{\left(1\right)}}}$ for y∈S(x) where i=1,2,…,10 and

j∈{1,2…..,32}. By using equation (8), through direct enumeration, we get the following 10 equations:

(20) $${v_{1}}^{\prime }=L_{2}{\left(a^{4}b^{4}c^{6}v_{1}+\frac{4\;{\left(a\;b\right)}^{2}}{v_{2}}+\frac{6}{c^{2}}\;v_{3}+\frac{4}{{\left(a\;b\right)}^{2}\;v_{4}}+\frac{c^{6}}{a^{4}b^{4}}v_{5}\right)}^{4}$$

(21) $$\begin{array}{c} {{\left(v_{2}^{\prime }\right)}^{-1}=L_{2}\left(a^{4}b^{4}c^{6}v_{1}+\frac{4\;{\left(a\;b\right)}^{2}}{v_{2}}+\frac{6}{c^{2}}\;v_{3}+\frac{4}{{\left(a\;b\right)}^{2}\;v_{4}}+\frac{c^{6}}{a^{4}b^{4}}v_{5}\right)}^{3}\\ \left(\frac{b^{4}c^{6}}{a^{4}v_{6}}+\frac{4\;b^{2}}{a^{2}}v_{7}+\frac{6}{{v_{8}\;c}^{2}}+\frac{4\;a^{2}}{b^{2}}v_{9}+\frac{a^{4}c^{6}}{b^{4}v_{10}}\right) \end{array}$$

(22) $$\begin{array}{c} {v_{3}^{\prime }=L}_{2}{\left(a^{4}b^{4}c^{6}v_{1}+\frac{4\;{\left(a\;b\right)}^{2}}{v_{2}}+\frac{6}{c^{2}}\;v_{3}+\frac{4}{{\left(a\;b\right)}^{2}\;v_{4}}+\frac{c^{6}}{a^{4}b^{4}}v_{5}\right)}^{2}\\ {\left(\frac{b^{4}c^{6}}{a^{4}v_{6}}+\frac{4\;b^{2}}{a^{2}}v_{7}+\frac{6}{{v_{8}\;c}^{2}}+\frac{4\;a^{2}}{b^{2}}\;v_{9}+\frac{a^{4}c^{6}}{b^{4}v_{10}}\right)}^{2} \end{array}$$

(23) $$\begin{array}{c} {\left({v_{4}}^{\prime }\right)}^{-1}={L_{2}\left(a^{4}b^{4}c^{6}v_{1}+\frac{4\;{\left(a\;b\right)}^{2}}{v_{2}}+\frac{6}{c^{2}}\;v_{3}+\frac{4}{{\left(a\;b\right)}^{2}\;v_{4}}+\frac{c^{6}}{a^{4}b^{4}}v_{5}\right)}^{2}\\ {\left(\frac{b^{4}c^{6}}{a^{4}v_{6}}+\frac{4\;b^{2}}{a^{2}}v_{7}+\frac{6}{{v_{8}\;c}^{2}}+\frac{4\;a^{2}}{b^{2}}v_{9}+\frac{a^{4}c^{6}}{b^{4}v_{10}}\right)}^{2} \end{array}$$

(24) $${v_{5}}^{\prime }=L_{2}\;{\left(\frac{b^{4}c^{6}}{a^{4}v_{6}}+\frac{4\;b^{2}}{a^{2}}v_{7}+\frac{6}{{v_{8}\;c}^{2}}+\frac{4\;a^{2}}{b^{2}}\;v_{9}+\frac{a^{4}c^{6}}{b^{4}v_{10}}\right)}^{4}$$

(25) $${\left({v_{6}}^{\prime }\right)}^{-1}=L_{2}\;{\left(\frac{a^{4}c^{6}}{b^{4}}v_{1}+\frac{4\;a^{2}}{b^{2}v_{2}}+\frac{6}{c^{2}}v_{3}+\frac{4\;b^{2}}{a^{2}v_{4}}+\frac{b^{4}c^{6}}{a^{4}}v_{5}\right)}^{4}$$

(26) $$\begin{array}{c} {v_{7}}^{\prime }={L_{2}\left(\frac{a^{4}c^{6}}{b^{4}}v_{1}+\frac{4\;a^{2}}{b^{2}v_{2}}+\frac{6}{c^{2}}v_{3}+\frac{4\;b^{2}}{a^{2}v_{4}}+\frac{b^{4}c^{6}}{a^{4}}v_{5}\right)}^{3}\\ \left(\frac{c^{6}}{a^{4}b^{4}v_{6}}+\frac{4}{a^{2}b^{2}}v_{7}+\frac{6}{c^{2}v_{8}}+4\;a^{2}b^{2}v_{9}+\frac{a^{4}b^{4}c^{6}}{v_{10}}\right) \end{array}$$

(27) $$\begin{array}{c} {\left({v_{8}}^{\prime }\right)}^{-1}=L_{2}{\left(\frac{a^{4}c^{6}}{b^{4}}v_{1}+\frac{4\;a^{2}}{b^{2}v_{2}}+\frac{6}{c^{2}}v_{3}+\frac{4\;b^{2}}{a^{2}v_{4}}+\frac{b^{4}c^{6}}{a^{4}}v_{5}\right)}^{2}\\ {\left(\frac{c^{6}}{a^{4}b^{4}v_{6}}+\frac{4}{a^{2}b^{2}}v_{7}+\frac{6}{c^{2}v_{8}}+4\;a^{2}b^{2}v_{9}+\frac{a^{4}b^{4}c^{6}}{v_{10}}\right)}^{2} \end{array}$$

(28) $$\begin{array}{c} {v_{9}}^{\prime }=L_{2}\left(\frac{a^{4}c^{6}}{b^{4}}v_{1}+\frac{4\;a^{2}}{b^{2}v_{2}}+\frac{6v_{3}}{c^{2}}+\frac{4\;b^{2}}{a^{2}v_{4}}+\frac{b^{4}c^{6}}{a^{4}}v_{5}\right)\\ {\left(\frac{c^{6}}{a^{4}b^{4}v_{6}}+\frac{4\;v_{7}}{a^{2}b^{2}}+\frac{6}{c^{2}v_{8}}+4\;a^{2}b^{2}v_{9}+\frac{a^{4}b^{4}c^{6}}{v_{10}}\right)}^{3} \end{array}$$

(29) $${\left({v_{10}}^{\prime }\right)}^{-1}=L_{2}{\left(\frac{c^{6}}{a^{4}b^{4}v_{6}}+\frac{4}{a^{2}b^{2}}v_{7}+\frac{6}{c^{2}v_{8}}+4\;a^{2}b^{2}v_{9}+\frac{a^{4}b^{4}c^{6}}{v_{10}}\right)}^{4}$$

From the system of equations ((20), (21), (22), (23), (24), (25), (26), (27), (28), (29)), we conclude that:

$${\left({v_{2}}^{\prime }\right)}^{-1}={\left({v_{1}}^{\prime }\right)}^{\frac{3}{4}}{\left({v_{5}}^{\prime }\right)}^{\frac{1}{4}},{v_{3}}^{\prime }={\left({v_{1}}^{\prime }\right)}^{\frac{1}{2}}{\left({v_{5}}^{\prime }\right)}^{\frac{1}{2}},{\left({v_{4}}^{\prime }\right)}^{-1}={\left({v_{1}}^{\prime }\right)}^{\frac{1}{4}}{\left({v_{5}}^{\prime }\right)}^{\frac{3}{4}}$$

$${v_{7}}^{\prime }={\left({v_{6}}^{\prime }\right)}^{-\frac{3}{4}}\;{\left({v_{10}}^{\prime }\right)}^{-\frac{1}{4}},{\left({v_{8}}^{\prime }\right)}^{-1}={\left({v_{6}}^{\prime }\right)}^{-\frac{1}{2}}{\left({v_{10}}^{\prime }\right)}^{-\frac{1}{2}},v_{9}={\left({v_{6}}^{\prime }\right)}^{-\frac{1}{4}}\;{\left({v_{10}}^{\prime }\right)}^{-\frac{3}{4}}$$

Therefore, we can only select the four independent real variables ${v_{1}}^{\prime },{v_{5}}^{\prime },{v_{6}}^{\prime }$ and ${v_{10}}^{\prime }\text{.}$

The consistency (computability) condition given in (6) is satisfied if the vector –valued boundary function h given in (19) is formed as:

(30) $$h\left(x\right)=\left(d,-\left(\frac{3\;d+e}{4}\right),\frac{d+e}{2},-\left(\frac{d+3e}{4}\right),e,n,-\left(\frac{3\;n+o}{4}\right),\frac{n+o}{2},-\left(\frac{n+3\;o}{4}\right),o\right)$$

where d,e,n,o∈R.

If we assume ${{u_{i}}^{4}=v}_{i}$, then the system of equations ((20), (21), (22), (23), (24), (25), (26), (27), (28), (29)) is reduced to:

(31) $${u_{1}}^{\prime }=\sqrt[{4}]{L_{2}}\left(\frac{4\;u_{1}{u_{5}}^{3}}{a^{2}b^{2}}+4\;a^{2}b^{2}{u_{1}}^{3}\;u_{5}+a^{4}b^{4}c^{6}{u_{1}}^{4}+\frac{6{u_{1}}^{2}{u_{5}}^{2}}{c^{2}}+\frac{c^{6}{u_{5}}^{4}}{b^{4}a^{4}}\right)\;{u_{5}}^{\prime }=\sqrt[{4}]{L_{2}}\;\left(\frac{6}{c^{2}{u_{6}}^{2}{u_{10}}^{2}}+\frac{b^{4}c^{6}}{a^{4}{u_{6}}^{4}}+\frac{a^{4}c^{6}}{b^{4}{u_{10}}^{4}}+\frac{4\;b^{2}}{a^{2}{u_{6}}^{3}u_{10}}+\frac{4\;a^{2}}{b^{2}u_{6}{u_{10}}^{3}}\right)$$

(32) $${\left({u_{6}}^{\prime }\right)}^{-1}=\sqrt[{4}]{L_{2}}\left(\frac{4\;b^{2}u_{1}{u_{5}}^{3}}{a^{2}}+\frac{4\;a^{2}{u_{1}}^{3}u_{5}}{b^{2}}+\frac{a^{4}c^{6}{u_{1}}^{4}}{b^{4}}+\frac{6{u_{1}}^{2}{u_{5}}^{2}}{c^{2}}+\frac{b^{4}c^{6}{u_{5}}^{4}}{a^{4}}\right)$$

(33) $${\left({u_{10}}^{\prime }\right)}^{-1}=\sqrt[{4}]{L_{2}}\;\left(\frac{6}{c^{2}{u_{6}}^{2}{u_{10}}^{2}}+\frac{c^{6}}{a^{4}b^{4}{u_{6}}^{4}}+\frac{a^{4}{b^{4}c}^{6}}{{u_{10}}^{4}}+\frac{4}{a^{2}b^{2}{u_{6}}^{3}u_{10}}+\frac{4\;a^{2}b^{2}}{u_{6}{u_{10}}^{3}}\right)$$

## 4 Translation-invariant Gibbs measures

Now, we describe the set of translation-invariant Gibbs measures corresponding to our model that is related to the Hamiltonian given in Ref. [2]. The vector-valued function

$$h\left(x\right)=\left\{h_{B_{1}\left(x\right),{\sigma_{j}}^{\left(1\right)}}:j\in \left\{1,2,.\;.\;.,32\right\}\right\}$$

is called a translation invariant one if $h_{B_{1}\left(x\right),{\sigma_{j}}^{\left(1\right)}}=h_{B_{1}\left(x^{\left(1\right)}\right),{\sigma_{j}}^{\left(1\right)}}$ , $\forall x^{\left(1\right)}\in S\left(x\right)\;\&\;j\in \left\{1,2,.\;.,32\right\}$, and the Gibbs measures that correspond to a translation-invariant boundary-function h are called translation-invariant Gibbs measures [4]. As a result, the Gibbs measure for our model is translation-invariant one if ${{u_{i}}^{\prime }=u}_{i}$, ∀i∈{1,5,6,10}.

Finding the set of all solutions of the system of equations (30), (31), (32), (33) is not so simple; therefore, we define a set $A_{1}$ as in Eq. (34) in which it is possible to solve the system:

(34) $$A_{1}=\left\{\left(u_{1},u_{5},u_{6},u_{10}\right)\in {R^{+}}^{4}:u_{5}=\frac{1}{u_{6}},\frac{1}{u_{10}}=u_{1}\right\}\;\text{.}$$

For simplicity, we introduce the following operator:

$$G\left(u\right):u\in {R^{+}}^{4}\;\to \left(G_{1}\left(u\right),G_{5}\left(u\right),G_{6}\left(u\right),G_{10}\left(u\right)\right)\in {R^{+}}^{4}$$

where $u=\left(u_{1},u_{5},u_{6},u_{10}\right)\in {R^{+}}^{4}$, $u_{1}^{\prime }=$ $G_{1}\left(u\right)$ , $u_{5}^{\prime }=G_{5}\left(u\right)$, ${u_{6}}^{\prime }=G_{6}\left(u\right)$ and ${u_{10}}^{\prime }=G_{10}\left(u\right)$.

The translation**₋**invariant Gibbs measures of the Ising-Vannimenus model corresponding to the Hamiltonian (2) are described by the positive fixed points of the operator G, i.e., the positive roots of the equation G(u)=u. It's clear that $G\left(A_{1}\right)\subseteq A_{1}$, i.e., the set $A_{1}$ is invariant under the operator G. Hence, we consider this invariant subset for this operator to describe the translation**₋**invariant Gibbs distributions.

### 4.1 Phase transitions

From [4], we know that there is more than one Gibbs measure corresponding to Hamiltonian [2] if there exist multiple positive fixed points of the defined operator G such that each point corresponds to a unique Gibbs measure. In essence, a phase transition occurs for Ising-Vannimenus model with Hamiltonian [2] if there is more than one solution to the system of equations (30), (31), (32), (33). The number of solutions to these equations depends on the coupling constants and the inverse temperature $\beta =\frac{1}{kT}>0$. One of the most interesting issues in statistical mechanics is to find an accurate value of temperature $T_{cr}$ such that a phase transition exists $\forall T\;<T_{cr}$, if it is possible to find this temperature then it is called the critical temperature of the model.

Now we start to analyze the system of equations (30), (31), (32), (33) in order to determine the number of positive fixed points for it. We restrict the operator G to the set $A_{1}$, i.e., let us assume $u_{5}=\frac{1}{u_{6}}$ and $u_{1}=\frac{1}{u_{10}}$. Then, by dividing equation (30) by (31), we reduce the nonlinear dynamical system of equations (30), (31), (32), (33) to the following equation:

$$\frac{u_{1}}{u_{5}}=\frac{\left(4c^{2}a^{2}b^{2}u_{1}{u_{5}}^{3}+4\;a^{6}b^{6}c^{2}{u_{1}}^{3}u_{5}+a^{8}b^{8}c^{8}{u_{1}}^{4}+6\;a^{4}b^{4}{u_{1}}^{2}{u_{5}}^{2}+c^{8}{u_{5}}^{4}\right)}{\left(6\;b^{4}a^{4}{u_{5}}^{2}{u_{1}}^{2}+b^{8}c^{8}{u_{5}}^{4}+a^{8}c^{8}{u_{1}}^{4}+4\;b^{6}a^{2}c^{2}u_{5}^{3}u_{1}+4\;a^{6}b^{2}c^{2}u_{5}{u_{1}}^{3}\right)}\text{.}$$

Assume $\frac{u_{1}}{u_{5}}=x,x>0\text{,}$ then we define the following function $f:R^{+}\to R^{+}$ such that:

$$f\left(x\right)=\frac{\left(a^{8}b^{8}c^{8}x^{4}+4\;a^{6}b^{6}c^{2}x^{3}+6\;a^{4}b^{4}x^{2}+4c^{2}a^{2}b^{2}x+c^{8}\right)}{\left(a^{8}c^{8}x^{4}+4\;a^{6}b^{2}c^{2}x^{3}+6\;b^{4}a^{4}x^{2}+4\;b^{6}a^{2}c^{2}x+b^{8}c^{8}\right)}$$

The equation f(x)=x coincides significantly with the results in [39] for Ising model with two competing interactions (nearest neighbors, and one-level next-nearest neighbors) on the Cayley tree of order four when $J_{p}=0$, i.e., b=1.

For simplicity, assume $a^{2}=a^{\prime },b^{2}=b^{\prime },c^{2}=c^{\prime }$. Then, the function f is reduced to:

(35) $$f\left(x\right)=\frac{\left({a^{\prime }}^{4}{b^{\prime }}^{4}{c^{\prime }}^{4}x^{4}+4\;{a^{\prime }}^{3}{b^{\prime }}^{3}c^{\prime }\;x^{3}+4{a^{\prime }b^{\prime }c}^{\prime }\;x+6\;{a^{\prime }}^{2}{b^{\prime }}^{2}x^{2}+{c^{\prime }}^{4}\right)}{\left({a^{\prime }}^{4}{c^{\prime }}^{4}x^{4}+4\;{a^{\prime }}^{3}b^{\prime }\;c^{\prime }\;x^{3}+6\;{b^{\prime }}^{2}{a^{\prime }}^{2}x^{2}+4\;a^{\prime }{b^{\prime }}^{3}c^{\prime }\;x+{b^{\prime }}^{4}{c^{\prime }}^{4}\right)}$$

The fixed points of f(x) are the roots of equation f(x)=x, the later could be reduced to:

(36) $$g\left(x\right)={a^{\prime }}^{4}{c^{\prime }}^{4}x^{5}+A\;x^{4}+Bx^{3}+Cx^{2}+D\;x-{c^{\prime }}^{4}=0$$

where $A=\left(4\;{a^{\prime }}^{3}b^{\prime }\;c^{\prime }-{a^{\prime }}^{4}{b^{\prime }}^{4}{c^{\prime }}^{4}\right)$, $B=\left(6\;{b^{\prime }}^{2}{a^{\prime }}^{2}-4\;{a^{\prime }}^{3}{b^{\prime }}^{3}c^{\prime }\right)$, $C=\left(4\;{{a^{\prime }b}^{\prime }}^{3}\;c^{\prime }-6\;{a^{\prime }}^{2}{b^{\prime }}^{2}\right)$ and $D=\left({b^{\prime }}^{4}{c^{\prime }}^{4}-4\;a^{\prime }\;b^{\prime }\;c^{\prime }\right)$.

Next, we consider all possible signs of the variables A,B,C and D and determine the number of sign changes of the polynomials g(x) and g(−x) in order to guess the possible number of positive and negative roots, respectively, using Descartes’ rule of signs [2]. See Table 2 below.

**Table 2:** The possible number of positive and negative zeroes of g(x) by using Descartes' rule.

A	B	C	D	Positive Roots	Negative Roots
+	+	+	+	1	0,2,4
+	+	+	–	1	0,2,4
+	+	–	+	Impossible^a^	
+	+	–	–	1 0,2,4	
+	–	–	+	Impossible	
+	–	+	–	1, 3	0,2
+	–	+	+	1,3	0,2
+	–	–	–	1	0,2,4
–	+	+	+	1, 3	0,2
–	+	+	–	Impossible	
–	+	–	+	Impossible	
–	+	–	–	Impossible	
–	–	–	+	1, 3	0,2
–	–	+	–	1,3	0,2
–	–	+	+	1, 3	0,2
–	–	–	–	1	0,2,4

a Impossible means this case is impossible to exist.

Since we are interested in the existence of more than one positive root of the polynomial g(x), we consider these cases from Table 2 above and search for the conditions on $a^{\prime },b^{\prime }$ and $c^{\prime }$ such that these cases are satisfied. For example, it is possible to find 3 positive roots of the function g(x) given in (36) if A>0,B<0,C>0 and D<0, and this condition is reduced to the inequalities in (37) below:

(37) $$\frac{27\;{a^{\prime }}^{3}}{8}<{b^{\prime }}^{3}{c^{\prime }}^{3}<\frac{4}{a^{\prime }}\;\&\;\frac{27}{8\;{a^{\prime }}^{3}}<{b^{\prime }}^{3}{c^{\prime }}^{3}<4\;a^{\prime }$$

We consider two cases in solving these inequalities.

1) If $a^{\prime }>1$, then the intersection of the inequalities in (37) is $\frac{27\;{a^{\prime }}^{3}}{8}<{b^{\prime }}^{3}{c^{\prime }}^{3}<\frac{4}{a^{\prime }}$ , but we must consider the condition $\frac{27\;{a^{\prime }}^{3}}{8}<\frac{4}{a^{\prime }}$ to be satisfied, so ${a^{\prime }}^{4}<\frac{32}{27}$ and hence $a^{\prime }<1.04339$. So, we conclude that the conditions for this case are:

$$1<a^{\prime }<1.04339\;\&\;\frac{3\;a^{\prime }}{2\;c^{\prime }}<\;b^{\prime }\;<\sqrt[{3}]{\frac{4}{a^{\prime }}}.\frac{1}{c^{\prime }},c^{\prime }>0\text{.}$$

2) If $a^{\prime }\leq 1\text{,}$ then in the same way as in Ref. [1], we conclude that the conditions for this case are:

$$0.958415\;<a^{\prime }\leq 1\;\&\;\frac{3}{2\;a^{\prime }\;c^{\prime }}<b^{\prime }<\sqrt[{3}]{\frac{4\;a^{\prime }}{{c^{\prime }}^{3}}},c^{\prime }>0\text{.}$$

In the same way, we can prove the other cases in Table 2 Thus, we conclude the following:

Remark: The maximum number of positive roots of the polynomial g(x) given in (36) corresponds to a translation**₋**invariant phases of the Ising-Vannimenus model with Hamiltonian (2) and boundary function h (3) is three based on Descartes' rule.

Although Descartes' rule is needed to confine the possible conditions for phase transition to exist, but to determine the exact conditions for phase transition, we need further analysis as following.

Proposition 1 *The equation*

(38) $$x=\frac{\left({a^{\prime }}^{4}{b^{\prime }}^{4}{c^{\prime }}^{4}x^{4}+4\;{a^{\prime }}^{3}{b^{\prime }}^{3}c^{\prime }\;x^{3}+6\;{a^{\prime }}^{2}{b^{\prime }}^{2}x^{2}+4{a^{\prime }\;b^{\prime }c}^{\prime }x+{c^{\prime }}^{4}\right)}{\left({a^{\prime }}^{4}{c^{\prime }}^{4}x^{4}+4\;{a^{\prime }}^{3}b^{\prime }\;c^{\prime }\;x^{3}+6\;{b^{\prime }}^{2}{a^{\prime }}^{2}x^{2}+4\;a^{\prime }{b^{\prime }}^{3}c^{\prime }\;x+{b^{\prime }}^{4}{c^{\prime }}^{4}\right)}$$

*with*$x>0,a^{\prime }>0,c^{\prime }>0$*and*$b^{\prime }>0$*has one positive solution if*$b^{\prime }<1$.

*If*$b^{\prime }>y\left(c^{\prime }\right)$*such that*$y\left(c^{\prime }\right)$≡*the positive root of the equation*:

$$-5{c^{\prime }}^{4}-12c^{\prime }\;y-6\;y^{2}+4c^{\prime }y^{3}+3{c^{\prime }}^{4}y^{4}=0,y>0\text{,}$$

*then*, *there exist*$\eta_{1}\left(a^{\prime },b^{\prime },c^{\prime }\right),\eta_{2}\left(a^{\prime },b^{\prime },c^{\prime }\right)>$ 0 *such that equation* (38) *has three positive roots if* $\eta_{1}\left(a^{\prime },b^{\prime },c^{\prime }\right)\;<\;1<\;\eta_{2}\left(a^{\prime },b^{\prime },c^{\prime }\right)\text{,}$ *where*

$${\;\eta }_{i}\left(a^{\prime },b^{\prime },c^{\prime }\right)=\frac{f\left(x_{pi}\right)}{x_{pi}}$$

*and* $x_{pi},i=1,2$ *are the positive solutions for the equation*:

$\left(5\;{a^{\prime }}^{4}{c^{\prime }}^{8}x^{4}-3{a^{\prime }}^{4}{b^{\prime }}^{8}{c^{\prime }}^{8}x^{4}+16{a^{\prime }}^{3}b^{\prime }{c^{\prime }}^{5}x^{3}\left(1+{a^{\prime }}^{2}x^{2}\right)-8{a^{\prime }}^{3}{b^{\prime }}^{7}{c^{\prime }}^{5}x^{3}\left(1+{a^{\prime }}^{2}x^{2}\right)+8a^{\prime }{b^{\prime }}^{3}c^{\prime }x\left({c^{\prime }}^{4}+6{a^{\prime }}^{2}x^{2}+6{a^{\prime }}^{4}x^{4}+{a^{\prime }}^{6}{c^{\prime }}^{4}x^{6}\right)+6{b^{\prime }}^{2}\left(3{a^{\prime }}^{2}{c^{\prime }}^{4}x^{2}+8{a^{\prime }}^{4}{c^{\prime }}^{2}x^{4}+3{a^{\prime }}^{6}{c^{\prime }}^{4}x^{6}\right)--2{b^{\prime }}^{6}\left(3{a^{\prime }}^{2}{c^{\prime }}^{4}x^{2}+8{a^{\prime }}^{4}{c^{\prime }}^{2}x^{4}+3{a^{\prime }}^{6}{c^{\prime }}^{4}x^{6}\right)+{b^{\prime }}^{4}\left(36{a^{\prime }}^{4}x^{4}+16{c^{\prime }}^{2}\left({a^{\prime }}^{2}x^{2}+{a^{\prime }}^{6}x^{6}\right)+{c^{\prime }}^{8}\left(1+{a^{\prime }}^{8}x^{8}\right)\right)\right)=0$*and*f(x)*is the function given in* [34].

*Proof*:

*Note that*f(x)*is continuous*∀x>0*,*$f\left(0\right)=\frac{1}{{b^{\prime }}^{4}}>0$*and*f*is a bounded function*, *as a result*, *the curve*y=f(x)*must intersect the line*y=x*at least once*. *This provides an element of the set of translation****₋****invariant Gibbs measures that correspond to the model*(2). *Thus*, *we conclude that there exists at least one Gibbs measure for our model*, *and we look for the existence of more*.

*Consider the first derivative of the function*f*in*(35):

$$f^{\prime }\left(x\right)=\frac{\alpha_{1}+\alpha_{2}+\alpha_{3}+\alpha_{4}+\alpha_{5}+\alpha_{6}}{\alpha }$$

*where*:

$$\alpha_{1}=4\;a^{\prime }{b^{\prime }}^{3}{c^{\prime }}^{5}\;\left({b^{\prime }}^{2}-1\right)+12\;{a^{\prime }}^{2}\;{b^{\prime }}^{2}\;{c^{\prime }}^{4}\;\left({b^{\prime }}^{4}-1\right)\;x$$

$$\alpha_{2}=12\;{a^{\prime }}^{3}\;b'\;c\;'\left(2\;{b^{\prime }}^{2}\;\left({b^{\prime }}^{2}-1\right)+{c^{\prime }}^{4}\;\left({b^{\prime }}^{6}-1\right)\right)\;x^{2}$$

$$\alpha_{3}=4\;{a^{\prime }}^{4}\;{c^{\prime }}^{2}\;\left({b^{\prime }}^{4}-1\right)\;\left(8\;{b^{\prime }}^{2}+{c^{\prime }}^{6}+{b^{\prime }}^{4}{c^{\prime }}^{6}\right)\;x^{3}$$

$$\alpha_{4}=12{a^{\prime }}^{5}b'c'\;\left(2{b^{\prime }}^{2}\left({b^{\prime }}^{2}-1\right)+{c^{\prime }}^{4}\left({b^{\prime }}^{6}-1\right)\right)x^{4}$$

$$\alpha_{5}=12{a^{\prime }}^{6}{b^{\prime }}^{2}{c^{\prime }}^{4}\left({b^{\prime }}^{4}-1\right)x^{5}$$

$$\alpha_{6}=4{a^{\prime }}^{7}{b^{\prime }}^{3}{c^{\prime }}^{5}\left({b^{\prime }}^{2}-1\right)x^{6}$$

$$\alpha ={\left({b^{\prime }}^{4}{c^{\prime }}^{4}+4a^{\prime }{b^{\prime }}^{3}c^{\prime }x+6{a^{\prime }}^{2}{b^{\prime }}^{2}x^{2}+4{a^{\prime }}^{3}b^{\prime }c^{\prime }x^{3}+{a^{\prime }}^{4}{c^{\prime }}^{4}x^{4}\right)}^{2}\text{.}$$

*It's clear that*$f^{\prime }\left(x\right)>0$ (*is increasing*) *if* $b^{\prime }>1,c^{\prime }>0,a^{\prime }>0$ δx>0, *but* f *is a decreasing function if* $b^{\prime }\;<\;1$ *and that means the existence of only one positive root of the equation* f(x)=x. *Thus*, *we can limit ourselves to the increasing case in which* $b^{\prime }\;>\;1\text{,}$ *i*. *e*, $\frac{J_{p}}{T}>0$ *and hence* $J_{p}>0\text{.}$

*Based on Preston proposition* [4], *we can find more than one solution for* f(x)=x *if and only if there exist more than one solution to the equation* $x\;f^{\prime }\;\left(x\right)=f\left(x\right)$, *that is reduced to the following*:

(39) $$\left(5\;{a^{\prime }}^{4}{c^{\prime }}^{8}x^{4}-3{a^{\prime }}^{4}{b^{\prime }}^{8}{c^{\prime }}^{8}x^{4}+16{a^{\prime }}^{3}b^{\prime }{c^{\prime }}^{5}x^{3}\left(1+{a^{\prime }}^{2}x^{2}\right)-8{a^{\prime }}^{3}{b^{\prime }}^{7}{c^{\prime }}^{5}x^{3}\left(1+{a^{\prime }}^{2}x^{2}\right)+8a^{\prime }{b^{\prime }}^{3}c^{\prime }x\left({c^{\prime }}^{4}+6{a^{\prime }}^{2}x^{2}+6{a^{\prime }}^{4}x^{4}+{a^{\prime }}^{6}{c^{\prime }}^{4}x^{6}\right)+6{b^{\prime }}^{2}\left(3{a^{\prime }}^{2}{c^{\prime }}^{4}x^{2}+8{a^{\prime }}^{4}{c^{\prime }}^{2}x^{4}+3{a^{\prime }}^{6}{c^{\prime }}^{4}x^{6}\right)-2{b^{\prime }}^{6}\left(3{a^{\prime }}^{2}{c^{\prime }}^{4}x^{2}+8{a^{\prime }}^{4}{c^{\prime }}^{2}x^{4}++3{a^{\prime }}^{6}{c^{\prime }}^{4}x^{6}\right)+{b^{\prime }}^{4}\left(36{a^{\prime }}^{4}x^{4}+16{c^{\prime }}^{2}\left({a^{\prime }}^{2}x^{2}{{+a}^{\prime }}^{6}x^{6}\right)+{c^{\prime }}^{8}\left(1+{a^{\prime }}^{8}x^{8}\right)\right)\right)=0\text{.}$$

*Using Mathematica*, *there are two positive roots of this equation if the following conditions are satisfied*:

*Let*$y\left(c^{\prime }\right)\equiv$*the positive root of equation* [39] *for a specific value of* $c^{\prime }>0$:

(40) $${-5c^{\prime }}^{4}-12c^{\prime }\;y-6\;y^{2}+4c^{\prime }y^{3}+3{c^{\prime }}^{4}y^{4}=0,y>0.$$

**Fig. 4:**
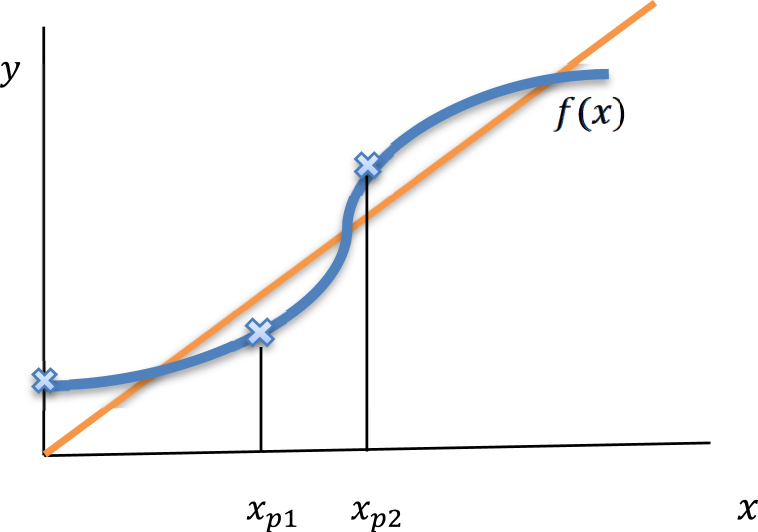
*There are three positive fixed points of the increasing bounded function*f(35)*if*f(0)>0, $f^{\prime }\left(x_{p1}\right)<1$*and*$f^{\prime }\left(x_{p2}\right)>1$.

Then, there are two positive zeros of the equation (39) if and only if $a^{\prime }\;>0,c^{\prime }>0$ $\delta \;b^{\prime }>y\left(c^{\prime }\right)\text{.}$ Assume that these conditions are satisfied, the roots of the polynomial in (39) are $x_{p1}$ *and* $x_{p2}$ *such that* ${0<x}_{p1}<\;x_{p2}\text{.}$ Then, there exits three positive fixed point of the function f(x) *if* $f^{\prime }\left(x_{p1}\right)<1<f^{\prime }\left(x_{p2}\right)$ , *i*.*e*, $f\left(x_{p1}\right)<x_{p1}$ *and* $f\left(x_{p2}\right)>x_{p2}$. The proof is completed (see Fig. 4**).**

*If the function*f*in equation*(35)*changes its concavity from up to down around*x=ν,*where*ν>0, *and if*$\eta_{1}\left(a',b',c'\right)=1$*or*$\eta_{2}\left(a',b',c'\right)=1$. *Then*, *there are exactly* 2 *positive roots of equation* (38). *Otherwise*, *if* $\eta_{1}\left(a',b',c'\right)<1<\eta_{2}\left(a',b',c'\right)\text{,}$ *then there are* 3 *positive roots* [11].

Theorem 1 *Ising-Vannimenus model with Hamiltonian* [2] *exhibits phase transitions if there exists more than one translation****₋****invariant Gibbs states corresponding to the positive zeros of the equation given in* [Ref. 38] *due to* proposition 1 *and the mentioned conditions*.

### 4.2 Numerical example

When T=24 and $J_{OL}=8.4$ , i.e, $c^{\prime }=2\text{,}$ then from proposition 4.1, there are two positive roots $x_{p1}$ and $x_{p2}$ of the equation (39) if $b^{\prime }\;>1.21224$ since the positive zero of the equation given in Ref. [39] is y(2)=1.21224.

Consider $b^{\prime }=1.4$, then there is a phase transition of the Vannimenus model that corresponds to Hamiltonian (2) if $f^{\prime }\left(x_{p1}\right)<1<f^{\prime }\left(x_{p2}\right)$ . This condition is satisfied if:

$$0.7906262026419042<a^{\prime }<1.264820210433788\;\text{.}$$

Let us take $a^{\prime }=0.8$, then the positive roots of the equation (39) are

$$x_{p1}=0.70685\;and\;x_{p2}=2.21050\;\text{.}$$

By proposition 4.1, we have three positive fixed points of the function f corresponding to three translation**₋**invariant Gibbs measures and these points are:

$$x_{1}=0.29259,x_{2}=1.96282\;and\;x_{3}=2.49463\text{.}$$

As it is known [36], if a is a fixed point for a continuously differentiable function f, then the dynamical system obtained by iterating the function f :

$$x_{n+1}=f\left(x_{n}\right),n=0,1,2,3,\ldots \text{.}$$

is stable at x=a if $\left|f^{\prime }\left(a\right)\right|<1$ and it is unstable if $\left|f^{\prime }\left(a\right)\right|>1\text{.}$

Now it's clear that $\left|f^{\prime }\left(x_{1}\right)\right|=0.17384<1$, $\left|f^{\prime }\left(x_{2}\right)\right|=1.19853>1$ and $\left|f^{\prime }\left(x_{3}\right)\right|=0.80809<1\text{.}$

**Fig. 5:**
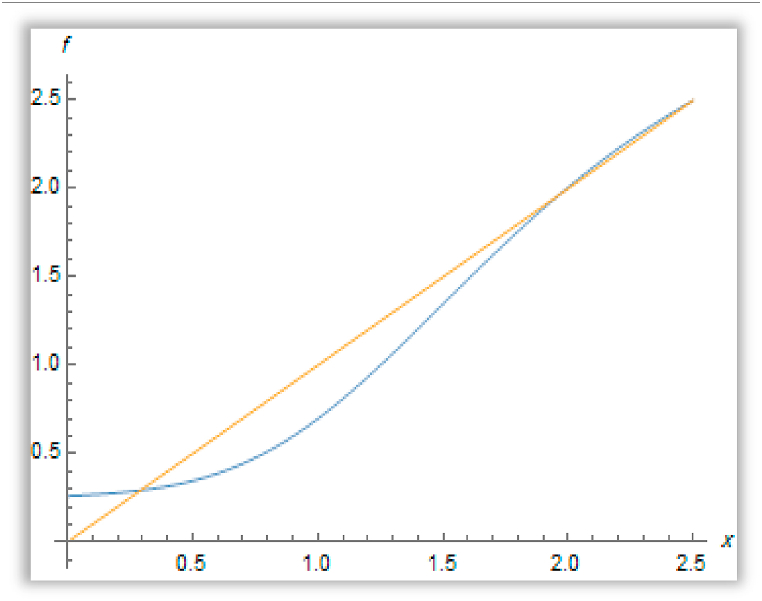
The existence of three positive fixed points for the function f(x) when $a^{\prime }=0.8,b^{\prime }=1.4\;\&c^{\prime }=2\text{.}$

So, we conclude that the first and third fixed points are stable, but the second one is unstable (Fig. 5).

The Gibbs measures $\mu_{1}$ and $\mu_{3}$ that corresponds to the stable fixed points $x_{1}$ and $x_{3}$ are called extreme ones [5] and [29].

for the function f(x) when $a^{\prime }=3.5,b^{\prime }=1.4$*&* $c^{\prime }=2\;\text{.}$

**Fig. 6:**
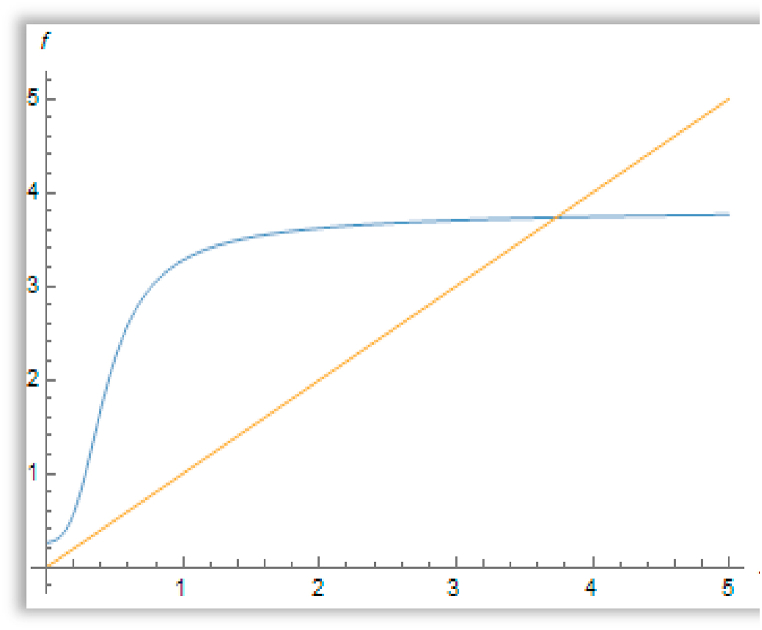
The existence of one positive fixed points.

In Fig. 6, there is only one positive fixed point for the function f given in Ref. [34] when $c^{\prime }=2$ , $b^{\prime }=1.4$ and $a^{\prime }=3.5$, but there exists two positive fixed points if $c^{\prime }=2$, $b^{\prime }=1.4$ and $a^{\prime }=0.7906262026419042$ since $f^{\prime }\left(x_{p2}\right)=1$ in this case. The positive fixed points of f(x) in this case are: {0.291872, 2.236709}.

**Fig. 7:**
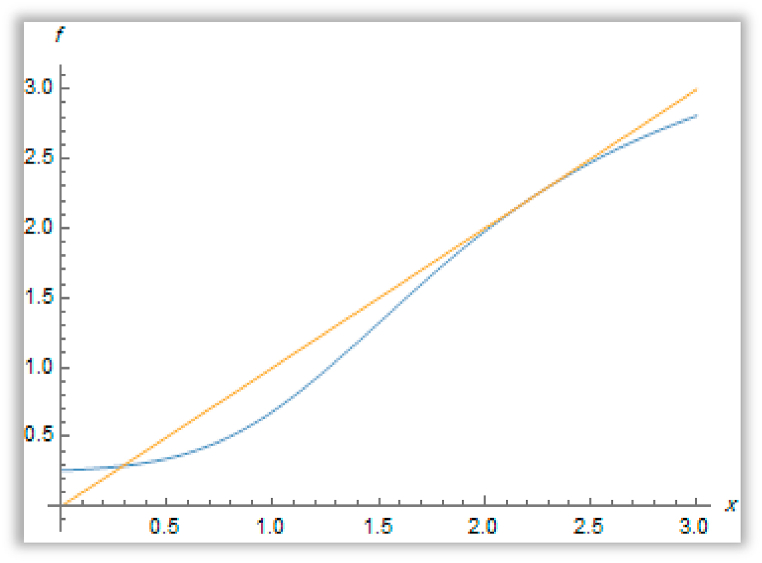
The existence of two fixed points for the function f(x) when $a^{\prime }=0.7906262026419042,b^{\prime }=1.4$*&*$c^{\prime }=2\text{.}$

Hence, we conclude that there exists two translation**₋**invariant Gibbs measures corresponding to these points (Fig. 7).

## 5 Conclusion and comments

Markov Random field method was used to obtain a set of translation**₋**invariant Gibbs states for Ising**₋**Vannimenus model with OLNNN interaction on fourth-order Cayley tree. This model was a development of Akin's work in Ref. [3] by adding one-level interaction to the case k=4. We have formed the set of recurrence equations associated to the model satisfying the consistency conditions (6). The existence of phase transition of the translation**₋**invariant measures by describing the positive fixed points of the recurrence equations (30), (31), (32), (33), was proved. The exact solution of this system of equations is not possible manually; so, we considered a specific case (38) and solved it. The conclusion was that if $J_{p}\;>0$, and under accurate conditions on J, $J_{OL}$ and the temperature T, it's possible to find three translation-invariant Gibbs measures, two of them are extreme ones. Finding the set of all Gibbs measures corresponding to the Hamiltonian (2) remains an interesting problem.

Comparing our results with the work in Ref. [3], one can notice that One-Level interaction has a considerable effect on phase transition conditions. So, we suggest adding this interaction to a Cayley tree of an arbitrary order as a future work.

Comparing our results with the results in Ref. [16], the existence of three competing interactions in our model has shown more precision in the conditions than the case of only two interactions. That accuracy is clearly shown in the numerical example, especially in the values of the parameter a.

Indeed, our model is an extension of Akin's work [3] for Cayley tree of order four (and other works) by adding one-level next-nearest-neighbor interaction.

Even though Cayley tree is not considered a realistic lattice, it's an amazing topology that helps to find exact solutions of various possible quantities. In addition, the problems on it are easier and simpler than d-dimensional integer lattices $\left(Z^{d}\right)$. Therefore, the results we have obtained can help in studying Ising models on the multidimensional lattices. Cayley tree of order 4 results can easily be generalized to the Rectangular Chandelier lattice in Ref. [13] with One-Level NNN interaction.

We have described some Gibbs measures with memory 2 corresponding to the Hamiltonian of our model (2), but there are still many problems related to this model, which are suggested as a possible future work. Some of these problems.

1. Defining the operator F on a different set and finding the corresponding Gibbs measures.
2. Finding the set of periodic Gibbs measures corresponding to the model (2). i.e., a description of such measures that correspond to the periodic fixed points of the defined operator.
3. Developing the model for a memory of length n, n>2

## Author contribution statement

**Sanabel M. Abu OUn:** Analyzed and interpreted the data; Contributed reagents, materials, analysis tools or data; Wrote the paper.

**Saed Mallak:** Conceived and designed the experiments; Performed the experiments; Analyzed and interpreted the data; Contributed reagents, materials, analysis tools or data.

**Jihad Asad:** Analyzed and interpreted the data; contributed reagents, materials.

## Funding statement

Prof. Saed Mallak, and Prof. Jihad Asad were supported by Palestine Technical University- Kadoorie [Scientific Research Fund 2023].

## Data availability statement

No data was used for the research described in the article.

## Declaration of interest’s statement

The authors declare no competing interests.
